# Involvement of Cyclooxygenase-2 in Establishing an Immunosuppressive Microenvironment in Tumorspheres Derived from TMZ-Resistant Glioblastoma Cell Lines and Primary Cultures

**DOI:** 10.3390/cells13030258

**Published:** 2024-01-30

**Authors:** Francesca Lombardi, Francesca Rosaria Augello, Serena Artone, Alessia Ciafarone, Skender Topi, Maria Grazia Cifone, Benedetta Cinque, Paola Palumbo

**Affiliations:** 1 Department of Life, Health and Environmental Sciences, University of L’Aquila, 67100 L’Aquila, Italy; francesca.lombardi@univaq.it (F.L.); francescarosaria.augello@univaq.it (F.R.A.); mariagrazia.cifone@univaq.it (M.G.C.); benedetta.cinque@univaq.it (B.C.); 2 PhD School in Medicine and Public Health, Department of Life, Health and Environmental Sciences, University of L’Aquila, 67100 L’Aquila, Italy; serena.artone@graduate.univaq.it; 3 PhD School in Health & Environmental Sciences, Department of Life, Health and Environmental Sciences, University of L’Aquila, 67100 L’Aquila, Italy; alessia.ciafarone@graduate.univaq.it; 4 Department of Clinical Disciplines, Aleksandër Xhuvani University, 3001 Elbasan, Albania; skender.topi@uniel.edu.al

**Keywords:** glioblastoma, COX-2, COXIB, temozolomide, tumor microenvironment, macrophages, osteopontin

## Abstract

Glioblastoma (GBM) is characterized by an immunosuppressive tumor microenvironment (TME) strictly associated with therapy resistance. Cyclooxygenase-2 (COX-2) fuels GBM proliferation, stemness, and chemoresistance. We previously reported that COX-2 upregulation induced by temozolomide (TMZ) supported chemoresistance. Also, COX-2 transfer by extracellular vesicles released by T98G promoted M2 polarization in macrophages, whereas COX-2 inhibition counteracted these effects. Here, we investigated the COX-2 role in the stemness potential and modulation of the GBM immunosuppressive microenvironment. The presence of macrophages U937 within tumorspheres derived from GBM cell lines and primary cultures exposed to celecoxib (COX-2 inhibitor) with or without TMZ was studied by confocal microscopy. M2 polarization was analyzed by TGFβ-1 and CD206 levels. Osteopontin (OPN), a crucial player within the TME by driving the macrophages’ infiltration, and CD44 expression was assessed by Western blot. TMZ strongly enhanced tumorsphere size and induced the M2 polarization of infiltrating macrophages. In macrophage-infiltrated tumorspheres, TMZ upregulated OPN and CD44 expression. These TMZ effects were counteracted by the concurrent addition of CXB. Remarkably, exogenous prostaglandin-E_2_ restored OPN and CD44, highlighting the COX-2 pivotal role in the protumor macrophages’ state promotion. COX-2 inhibition interfered with TMZ’s ability to induce M2-polarization and counteracted the development of an immunosuppressive TME.

## 1. Introduction

Glioblastoma (GBM) is a devastating disease characterized by a unique tumor microenvironment (TME) bearing a poor prognosis and relapse [1]. Many of the latest approaches have failed to improve outcomes; thus, new targeted therapies are desperately needed [2,3]. An extensive understanding of TME physiology is a critical issue in helping the development of effective treatments. TME is a “complex integrated system formed by the interaction of tumor cells with surrounding tissues and immune cells” [4]. The GBM microenvironment is enriched in neoplastic and non-neoplastic cells, such as tissue-resident cell types, resident microglia cells, and the newly recruited immune cells. The macrophages infiltrating the tumor, “glioblastoma-associated macrophages” (GAMs), represent the major cellular immune component adopting different activation states (pro-inflammatory “M1” and anti-inflammatory/pro-tumoral “M2” phenotypes) [5] and ably influence the TME secreting soluble mediators [6,7,8]. Despite the abundance of immune cells, the TME is a highly immunosuppressive due to M2-like GAMs secreting immunosuppressive factors (i.e., IL-6, TGF-β, IL-10) contributing to immune evasion [7].

In the context of the cellular heterogeneity of the TME, GBM stem cells (GSCs) account for a small population showing self-renewal, multilineage differentiation, and high resistance to conventional therapy [9]. In the TME, the physiological heterotypic interaction between GAMs and GBM cells, including GSCs, promoting recruitment of additional macrophages and the suppression of CD4+ and CD8+T cell infiltration and activity, actively sustains the tumor cell proliferation, invasion, angiogenesis, and stemness potential [10,11]. Specifically, secreted molecules from GSCs can induce the recruitment and polarization of GAMs, which in turn, sustain GSCs’ self-renewal by secreting stemness-supporting factors [10].

Osteopontin (OPN), a secreted multifunctional phosphorylated glycoprotein, plays a crucial role within the TME in several physiological and pathological processes, including macrophage recruitment and polarization, cell growth, and angiogenesis [12,13,14]. OPN is expressed in various immune cells and plays a role in initiating immune responses [15,16]. OPN is upregulated within glioblastoma-infiltrating neutrophils and macrophages and is associated with the infiltration of these cells within tumor specimens [17]. Moreover, the high OPN expression in GBM correlates positively with the grade and GAM infiltration and negatively with patient prognosis [14,18,19]. Furthermore, OPN acts as a significant regulator of GSC phenotype. The OPN stable knockdown impairs the sphere formation in U87MG, U251MG, T98G and LN18, GBM cell lines downmodulating the main stemness transcription factors and EGFR activation [20,21].

OPN activity in the TME is favored by its association with cell surface receptors such as integrins and CD44, a hyaluronan transmembrane receptor and a well-established GBM stem cell marker [22]. The OPN–CD44 interaction triggers the CD44 cleavage and the release of its intracellular domain (CD44-ICD) that translocates into the nucleus and, enhancing the expression of hypoxia-inducible factor, modulates the GBM hypoxic microenvironment [23,24]. The critical role of the OPN–CD44 interaction in maintaining the stemness phenotype has been shown [20]. Cells with a variant OPN construct lacking a C-terminal domain responsible for interactions with CD44 were not able to generate spheres [20].

In the TME, OPN is associated with chemoresistance in several cancers [13]. The TMZ, a DNA alkylating agent used as a standard first-line treatment for adult GBM patients, significantly enhanced the OPN expression and NF-κB activation in human U251MG cells. Moreover, the OPN silencing restored the TMZ sensitivity by blocking the NF-κB and Bcl-2 expression induced by TMZ [25].

Recently, we studied the influence of COX-2 on TMZ resistance, demonstrating the ability of TMZ to significantly upregulate COX-2 expression and pathways involved in the GBM-chemoresistance in TMZ-resistant GBM cells. Of note, the COX-2 inhibitor counteracted the TMZ action, demonstrating that the effects on T98G cells are owing to TMZ-induced COX-2 upregulation [26]. We have also reported that TMZ induced the COX-2 transfer by extracellular vesicles from T98G to human recipient macrophages, promoting the M2 phenotype polarization. Interestingly, the treatment with the selective COX-2 inhibitor, NS398, concurrent with TMZ, overcame the TMZ-induced overexpression of β-catenin, O-6-methylguanine-DNA methyltransferase (MGMT), and SOX-2 in T98G and lowered the levels of COX-2 shuttled in extracellular vesicles. These data confirmed the crucial role of the COX-2/PGE2 system in the cascade of events activated by TMZ and implicated in GBM chemoresistance [27].

To further elucidate the role of COX-2 in the TMZ resistance of GBM, here we investigated the potential ability of TMZ-induced COX-2 to influence the stemness potential evaluated through GBM-sphere generation, a three-dimensional (3D) model which, better than 2D models, considers the tumor complexity. A COX-2 inhibitor, celecoxib (CXB), alone or combined with TMZ, was used on T98G (TMZ-resistant) and U87MG (TMZ-partially resistant) [28] and GBM primary cultures. The heterotypic interaction of human macrophage cell line U937 with tumorspheres derived from treated GBM cells has been studied to define the COX-2 role in the modulation of the TME. Given the crucial role played by OPN in macrophage recruitment, we also verified whether TMZ could affect OPN levels and if this process was modulated by COX-2 inhibition.

## 2. Materials and Methods

### 2.1. Cell Lines

Human GBM cell lines, T98G and U87MG, were acquired from the European Collection of Authenticated Cell Cultures (ECACC,T98G: ECACC 92090213, U87MG: ECACC 89081402); human monocyte cell line, U937 were acquired from Cell Lines Service (Eppelheim, Germany). U937 cells are intensely used in macrophage–GBM cell interaction because of their ability to mimic the macrophage differentiation process [29,30,31]. T98G and U87MG cells were cultured according to manufacturer recommendations in Dulbecco’s Modified Eagle’s Medium (DMEM) supplemented with 10% (*v*/*v*) of fetal calf serum (FCS), 2 mM L-glutamine, 100 U/mL penicillin, and 100 mg/mL streptomycin (complete medium) (EuroClone, West York, UK). U937 cells were cultured in RPMI-1640 medium (EuroClone, West York, UK) supplemented with 10% (*v*/*v*) of FCS, 2 mM L-glutamine, 100 U/mL penicillin, and 100 mg/mL streptomycin (complete medium). All cells were maintained at 37 °C in 5% CO_2_ and 95% humidity, and media were totally replaced every 3 days. To induce the in vitro differentiation of U937 into a macrophage-like phenotype (M0), cells were incubated with 100 ng/mL phorbol 12-myristate 13-acetate (PMA, Sigma-Aldrich, Saint Louis, MO, USA) for 48 h as previously reported [32].

T98G cells express high levels of MGMT (“TMZ-resistant”) and are COX-2-positive cells; U87MG, COX-2-positive cells, do not express MGMT (“TMZ-partially resistant”) [28]. Cell number and viability were assayed by trypan blue staining under microscopy (Eclipse 50i, Nikon Corporation, Tokyo, Japan).

### 2.2. GBM Primary Cultures

Resected tissues from two GBM patients were obtained from the Operative Unit of Neurosurgery at the San Salvatore Hospital of L’Aquila. Each patient signed a written consent in accordance with the approved ethical permit from the regional ethics Internal Review Board (20 January 2015). Primary cell cultures (GL25 and GL44) were generated from fresh resected tumors, clinically and histologically characterized as GBM, and frozen at early passages as previously described [33]. Thawed primary cultures before use were characterized for stemness properties by immunofluorescence staining after NeuroCult™ differentiation medium incubation (Stem cell Technologies, Vancouver, BC, Canada). In particular, the cells were differentiated in the three neural lineages (neurons, astrocytes, and oligodendrocytes) through the specific media for 35 days. Supplementary Figure S1 shows representative images of GL25 and GL44 cells differentiated into astrocytes, neurons, and oligodendrocytes. Both the primary cultures were able to differentiate into a larger amount of GFAP+ astrocytes, NF200+ neurons, and a few OP4+ oligodendrocytes, confirming their stemness potential (Supplementary Figure S1).

Basal COX-2 expression was evaluated in primary cultures in adherent and tumorsphere conditions, and the results of representative Western blotting and relative densitometric analysis are shown (Supplementary Figure S2). Both cultures express COX-2 at higher levels in tumorspheres with respect to adherent cells.

### 2.3. Tumorsphere Formation Assay

The tumorsphere formation assay is a widely used method to obtain putative CSCs. For tumorsphere generation, all adherent cultures (5 × 10^5^ cells/well) were grown in ultra-low attachment plates in serum-free DMEM/F12 (1:1, vol/vol) with B27-reagent (Thermo Fisher Scientific, Waltham, MA, USA), EGF and FGF-β (both 20 ng/mL) (ImmunoTools GmbH, Friesoythe, Germany), penicillin/streptomycin, and glutamine (tumorspheres’ complete medium). Media were replaced every 3 days until sphere formation (~7 days) [34]. The morphology was detected and analyzed by microscope, Nikon Eclipse TS100, and area was assessed using ImageJ software 1.54d. Briefly, 10 bright field images at 4× magnification were randomly taken from all cells and analyzed. The tumorsphere average area (total area/number of tumorspheres) was expressed in mm^2^ (Supplementary Figure S3).

### 2.4. Reagents and Treatments

The selective COX-2 inhibitor, celecoxib (CXB) (Sigma-Aldrich, Saint Louis, MO, USA) was stored in a stock solution in DMSO at −20 °C and diluted in complete culture medium before use. Temozolomide (TMZ) (Sigma-Aldrich) was dissolved in 10% dimethylsulphoxide (DMSO) (stock solution of 51.5 mM). Working concentrations were daily prepared in PBS. Based on our previous report [26] and other GBM in vitro studies [35,36], for both adherent cell lines, we choose the concentrations of CXB 50 µM and TMZ 200 µM. The drugs were used as single agents or combined and were added simultaneously. Cells treated with DMSO alone (vehicle) were used in all the experiments as the “control” (not treated, CNTR). After 72 h treatment, the cells were counted, and 5 × 10^5^ of treated and not-treated cells were grown in tumorsphere complete medium in the presence of macrophages (70,000 cells) until sphere generation.

To evaluate the effects of exogenous PGE2 (Sigma-Aldrich) on GBM cells, cells were plated at 5000 cells/ cm^2^, left to adhere, and then simultaneously incubated with CXB and TMZ, as previously described, and with PGE2 (10 µM) for 72 h [37].

### 2.5. Proliferation Assay

The proliferation of primary cultures exposed to increasing concentrations of TMZ (10–400 µM) or the drug vehicle DMSO (CNTR) (72 h) was evaluated by cell-counting kit-8 (CCK-8). Absorbance at 450 nm was detected using a microplate reader (BioRad, Hercules, CA, USA). Data were expressed as optical density values (OD). The concentrations of TMZ ranged between 10 and 200 μM did not affect the cell viability of both cultures (Supplementary Figure S4). The higher concentration (400 μM) significantly reduced it to less than 50% in both primary cultures. The concentration of 200 μM TMZ, able to maintain the proliferation at 72 h above 50%, was chosen for both primary cultures. Similar to T98G, both primary cultures can be defined “TMZ-resistant”.

### 2.6. Macrophage Infiltration into Tumorspheres

To detect the presence of U937 within tumorspheres, the macrophages were alone labeled with the fluorescent lipophilic dye PKH26 that stably integrates into the cell membrane (Sigma-Aldrich). Briefly, 10^6^ macrophages mL^−1^ were centrifuged for 5 min. Pellets were resuspended with 1 mL of Diluent C. Then, 4 μL of PKH26 was added to the cell suspension. After incubation of the cell/dye suspension for 5 min, the staining was stopped by adding 2 mL of serum. Cells were washed following two more centrifugation steps (400× *g* for 10 min) to ensure the removal of unbound dye and then resuspended in a complete medium. The PKH26-labeled macrophages were incubated with GBM cell suspensions previously treated with CXB and TMZ. Cocultures of tumorspheres and macrophages were left to adhere overnight on coverslips pre-coated with poly-lysine (30 µg/mL) (Sigma-Aldrich). Coverslips mounted with Vectashield Mounting Medium (Vector Laboratories, Inc., Newark, CA, USA) were examined with a Leica TCSSP5 confocal microscope (Leica, Wetzlar, Germany). Z-stack images were generated and analyzed with Leica TCSSP5 confocal microscope software LAS-AF. Red fluorescent spots (3 fields/condition) were analyzed by the image processing tool of ImageJ software calculating the “corrected total cell fluorescence” (CTCF) = integrated density – (area of selected cell × mean fluorescence of background readings). For each image, three background areas were used to normalize against autofluorescence.

### 2.7. Western Blot

Cells were collected in ice-cold RIPA buffer (Merck KGaA, Darmstadt, Germany) containing a 100 mM protease inhibitor cocktail (Sigma-Aldrich). Protein concentration was determined by a BioRadTM BCA Protein Assay Kit (BioRad). Total cell lysates (25 μg protein/lane) were separated by 10% SDS-PAGE in reducing conditions with β-mercaptoethanol 5%. Proteins were electroblotted onto 0.45 µm nitrocellulose membranes (BioRad). Following incubation with 5% non-fat dry milk in Tris-buffered saline for 1h at room temperature, the membranes were incubated overnight at 4 °C with primary antibodies (Table 1). As secondary antibodies, peroxidase-conjugated anti-rabbit and anti-mouse IgG antibodies (dilution 1:2000) were acquired from Sigma-Aldrich. The ECL (Amersham Pharmacia Biotech, Buckinghamshire, UK) was used according to the manufacturer’s instructions to detect chemiluminescent signals. Emission was captured using the chemiluminescence documentation system ALLIANCE (UVITEC, Cambridge, UK).

### 2.8. ELISA Kit

TGFβ-1, IL-10, IL-1β, and OPN levels were quantified in the supernatants of tumorspheres by an enzyme-linked immunosorbent assay (ELISA) (Sigma-Aldrich). The supernatants were centrifuged at 1000× *g* (15 min). All the concentrations were determined by comparison to a standard curve. Results are expressed as pg/mL.

### 2.9. Flow Cytometry Analysis

M2-like macrophages were identified as CD206-positive [38]. Cell suspensions of GBM-spheres were dissociated by Accutase solution to obtain a single cell suspension [39], incubated with BSA 2% (10 min), and stained with a monoclonal mouse APC-conjugated CD206 antibody (BD Biosciences, San José, CA USA) or with the APC Mouse IgG1, κIsotype Control (BD Biosciences). The histograms of the CD206 fluorescence signal were obtained from gated events with the forward and side light-scatter characteristics of the dissociated cell populations. As a negative control, the U937 macrophage cell line (M0) without the addition of GBM cells was used. Fluorescence was measured using a FACSCanto™ II flow cytometer and FACSDiva software v6.1.3.

### 2.10. Statistics Analysis

Statistical analysis was performed while using GraphPad Prism 6.01 (GraphPad Software, San Diego, CA, USA). A Student’s unpaired t-test was used to compare the two means. The data were also evaluated using a one-way ANOVA test followed by a Tukey’s post hoc test. Data were from independent experiments repeated two or three times and performed in duplicate or triplicate. The results were shown as the means ± SD (standard deviation) or means ± SEM (standard error mean). *p* values less than 0.05 were considered significant.

## 3. Results

### 3.1. Effect of CXB, TMZ, and Their Combination on Tumorsphere Formation and Macrophage Infiltration

Adherent cell lines and primary cultures were exposed at the same time to CXB and TMZ as single drugs or in a drug-combination approach for 72h. Then, an equal number of treated cells (5 × 10^5^) were cultured in GSC medium in the presence of macrophages until tumorsphere formation. Phase contrast images showed that all the not-treated (CNTR) cells generated tumorspheres, although of different sizes. The CXB treatment slightly modified the spheres’ size; conversely, TMZ significantly increased it when compared to relative CNTR (Supplementary Figure S3), confirming the results of Gao et al. [40]. The drug combination (CXB+TMZ) treatment hindered the formation of tumorspheres, which appeared smaller and irregular compared to other treatments (Supplementary Figure S3).

**Figure 1:**
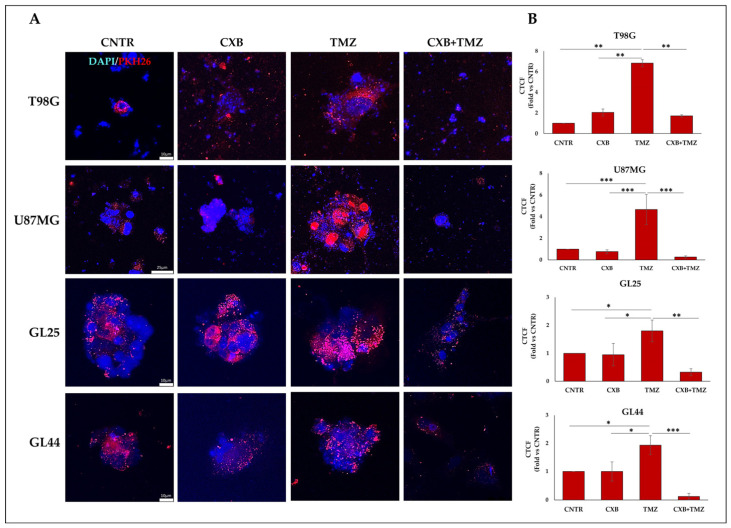
Detection of human macrophage cell line U937 in GBM spheres by confocal microscopy. (**A**) U937 were stained with PKH26 Red Fluorescent Cell Linker and cultured with adherent GBM cells previously exposed for 72 h to CXB, TMZ, or their combination until tumorsphere formation. Representative Z-stack projections of tumorspheres from T98G, U87MG, and primary cultures (GL25, GL44) after infiltration of PKH26-labeled macrophages (red) are shown. Dapi dye (blue) was used to counterstain nuclei. Images are from one of two independent experiments (magnification 63×). (**B**) Quantification of tumorsphere-infiltrating macrophages. For the quantification of the red fluorescence of PKH26-labeled macrophages, digital images were analyzed by ImageJ software. The red fluorescence intensity was expressed as the mean values of CTCF (corrected total cell fluorescence) ± SD and are expressed as the fold change vs. CNTR. A one-way ANOVA with a Tukey’s post hoc test was applied (* *p* < 0.05, ** *p* < 0.01, *** *p* < 0.001).

The presence of the human macrophage U937 within GBM spheres was verified following red fluorescence PKH26 staining by confocal immunofluorescence images (Figure 1A). To assess the tumorsphere infiltration, the macrophages were previously labeled with PKH26 (red spots), and the red fluorescence, quantified by ImageJ calculating the corrected total cell fluorescence (CTCF), indicated that macrophages were effectively internalized in GBM spheres (Figure 1B). Overall, tumorsphere-infiltrating macrophages were significantly higher in TMZ-treated cultures than CNTR in cell lines and primary cultures (Figure 1B). The red fluorescence was considerably lower than control levels after CXB+TMZ treatment in all cell cultures, suggesting that the drug combination harmfully affected both tumorsphere formation and macrophage infiltration (Figure 1B).

### 3.2. COX-2 Inhibition Affects the Immunosuppressive Macrophage M2 Phenotype

**Figure 2:**
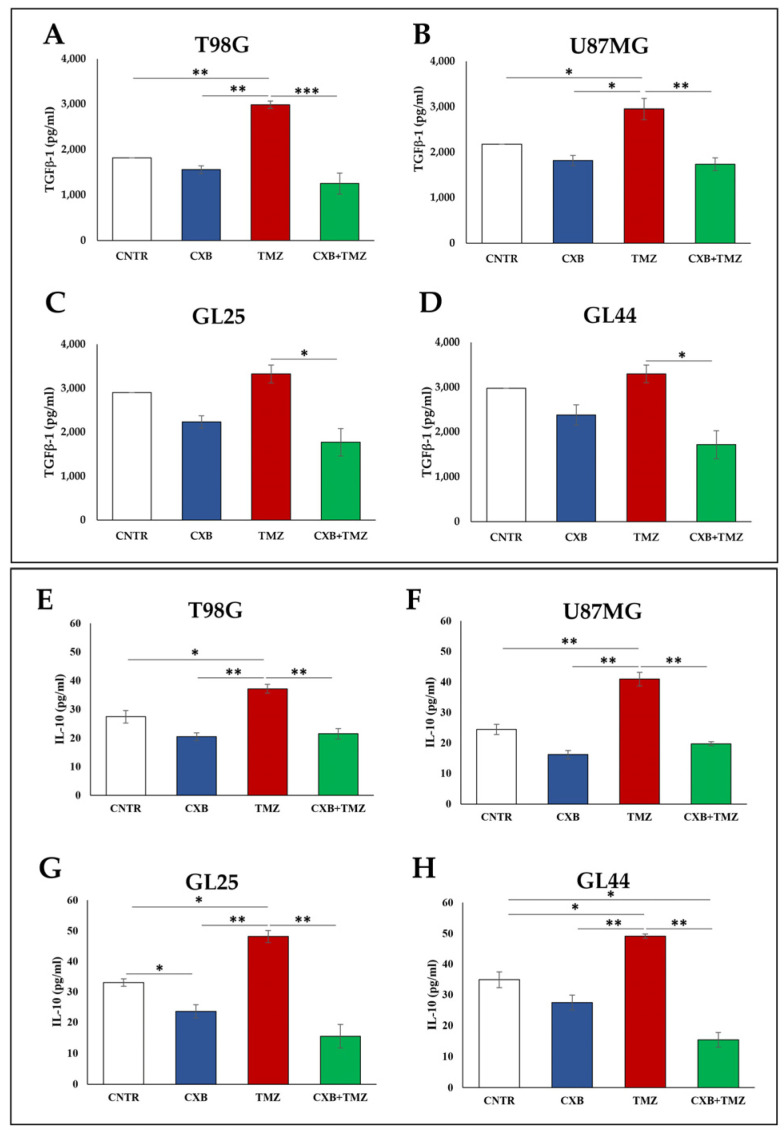
M2-phenotypic shift after macrophage–tumorsphere interaction. GBM cells were treated with CXB, TMZ, and a drug combination for 72 h, then cultured in tumorsphere medium with macrophages. The levels of TGF-β1 and IL-10, M2-related markers, were assessed in coculture supernatants by ELISA (**A**–**D** and **E**–**H**, respectively). Data from three experiments are expressed as the mean ± SEM. A one-way ANOVA with a Tukey’s post hoc test was used (* *p* < 0.05, ** *p* < 0.01, *** *p* < 0.001).

The phenotype of tumorsphereinfiltrating macrophages has been analyzed. GBM cells previously exposed or not (CNTR) to CXB, TMZ, or their combination were cocultured with macrophages until the tumorspheres’ generation. To verify the M2 polarization, TGF-β1, the most common M2-related marker, was assayed in supernatants. Figure 2A–D shows the TGF-β1 levels released by cell cultures in the presence of macrophages. The COX-2 inhibition did not cause a TGF-β1 modulation; otherwise, the TMZ exposure increased TGF-β1 levels in all cell cultures, being significant in T98G and U87MG compared to CNTR (Figure 2A,B). Of note, the drug combination CXB+TMZ induced a relevant lowering of TGF-β1 in the supernatants of T98G, U87MG, GL25, and GL44 cells relative to TMZ (Figure 2A–D). Interleukin 10 (IL-10), another immunosuppressive cytokine and M2 marker, was also assayed in the same samples. As for TGF-β1, the results (Figure 2E–H) show that, in all cells, TMZ alone induced a significant increase in IL-10 levels, which were strongly reduced in the presence of the CXB + TMZ combination.

**Figure 3:**
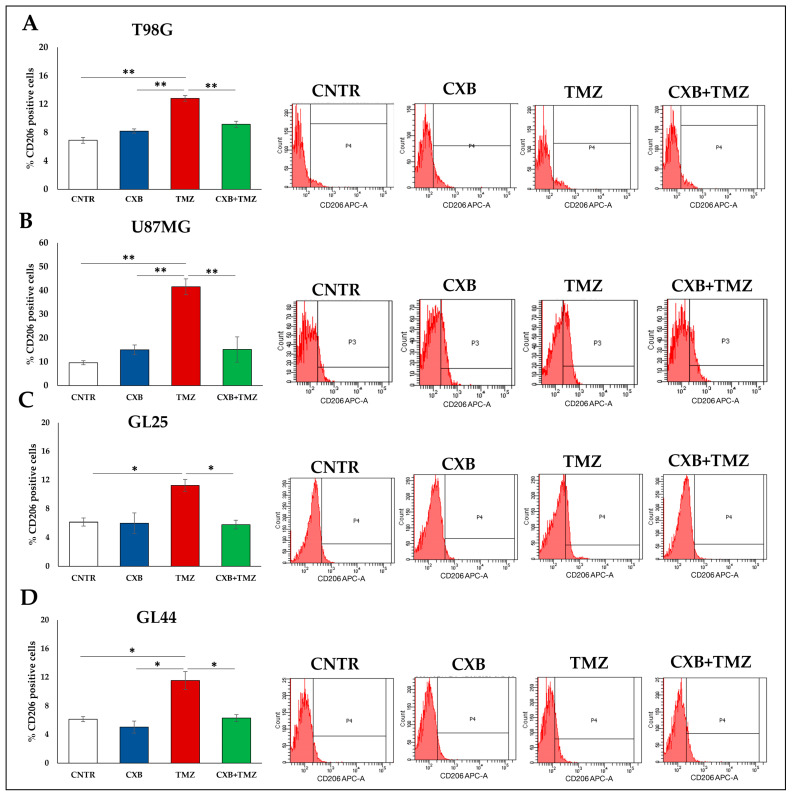
CD206 detection by flow cytometry. GBM cells were treated with CXB, TMZ, and a drug combination for 72 h, then cultured in tumorsphere medium with macrophages. The M2-differentiation marker, CD206, was evaluated by flow cytometry, and data were expressed as the percentage of CD206-positive cells (**A**–**D**). Data from two experiments are expressed as the mean ± SEM. One-way ANOVA with Tukey post-hoc test was used (* *p* < 0.05, ** *p* < 0.01). Flow cytometric profiles of CD206 positive cells from one representative experiment are also shown.

To further confirm the M2 polarization state of macrophages that infiltrated into GBM tumorspheres, the surface expression of the M2-differentiation marker, CD206, was also analyzed by flow cytometry (Figure 3A–D). Overall, TMZ remarkably upregulated CD206 in U937-infiltrated tumorspheres, while the drug combination significantly counteracted the TMZ effect reducing CD206 levels (Figure 3A–D).

**Figure 4:**
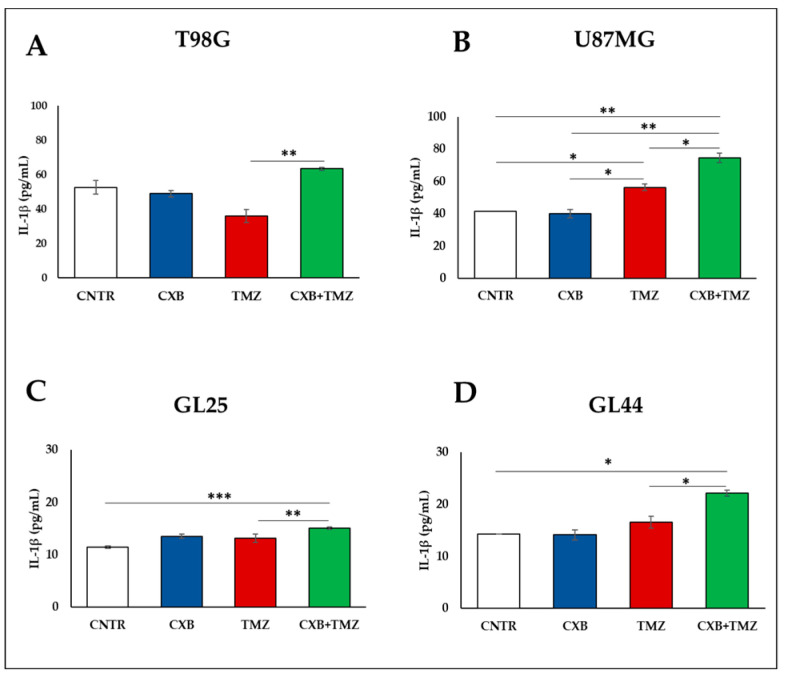
Concentration levels of IL-1β, M1-related marker, after interaction between macrophages and tumorspheres were assessed by ELISA. T98G (**A**), U87MG (**B**) cell lines and GL25 (**C**) and GL44 (**D**) were treated with CXB, TMZ, or their combination for 72 h, then cultured in GSC medium with macrophages. Data from three experiments in duplicate are expressed as the mean ± SEM. A one-way ANOVA with a Tukey’s post hoc test was used (* *p* < 0.05, ** *p* < 0.01, *** *p* < 0.001).

The content of interleukin-1β (IL-1β), a cytokine associated with the M1-activation state, was also measured in the supernatants of cell lines and primary cultures exposed to CXB, TMZ, or their combination in the presence of macrophages. CXB alone did not change IL-1β levels, while, when combined with TMZ, it could significantly increase the IL-1β secretion from all cells with respect to TMZ (Figure 4A–D).

All these findings suggested that TMZ caused a macrophage M2 phenotype shift following interaction with GBM cells, and the drug combination effectively counteracted the action of TMZ.

### 3.3. COX Inhibition Counteracted TMZ-Induced OPN Overexpression

**Figure 5:**
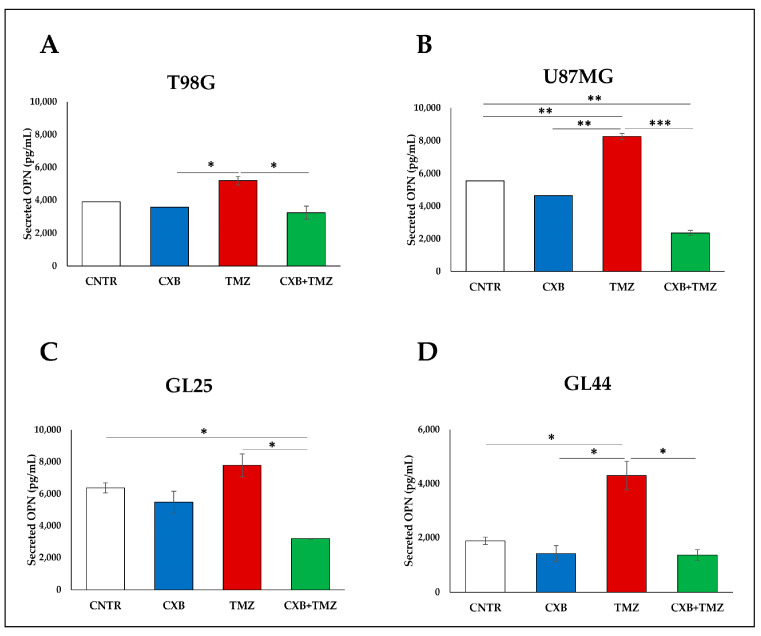
Levels of OPN released by the GBM-sphere/macrophage coculture. U87MG, T98G, GL25, and GL44 were treated or not (CNTR) with CXB, TMZ, or their combination for 72 h; treated cells were cultured in GSC medium until sphere formation in the presence of macrophages. OPN levels were analyzed by ELISA in the supernatants of GBM spheres generated after interaction with macrophages (**A**–**D**). Results from two experiments in duplicate are expressed as the mean ± SEM. A two-way ANOVA with a Tukey’s post hoc test was used (* *p* < 0.05, ** *p* < 0.01; *** *p* < 0.001).

OPN is generally expressed both by GBM cells and macrophages [11]. The amount of OPN secreted in supernatants of CXB-, TMZ-, or CXB+TMZ-treated cells in the presence of U937 is shown in Figure 5. The basal OPN expression was higher in U87MG than in T98G cells and GBM primary cultures, thus confirming previous evidence [20]. In all cell systems, the TMZ exposure enhanced the OPN release compared to CNTR, CXB, and the drug combination treatment (Figure 5A–D). Of note, CXB, which alone did not significantly influence the OPN levels, when added together with TMZ, counteracted the TMZ-induced OPN secretion in both cell lines and primary cultures (Figure 5A–D). The effect of CXB, when combined with TMZ, could be due to its ability to inhibit the TMZ-induced COX-2, involved in the OPN upregulation, thus allowing TMZ to perform its actions optimally, such as the OPN reduction.

**Figure 6:**
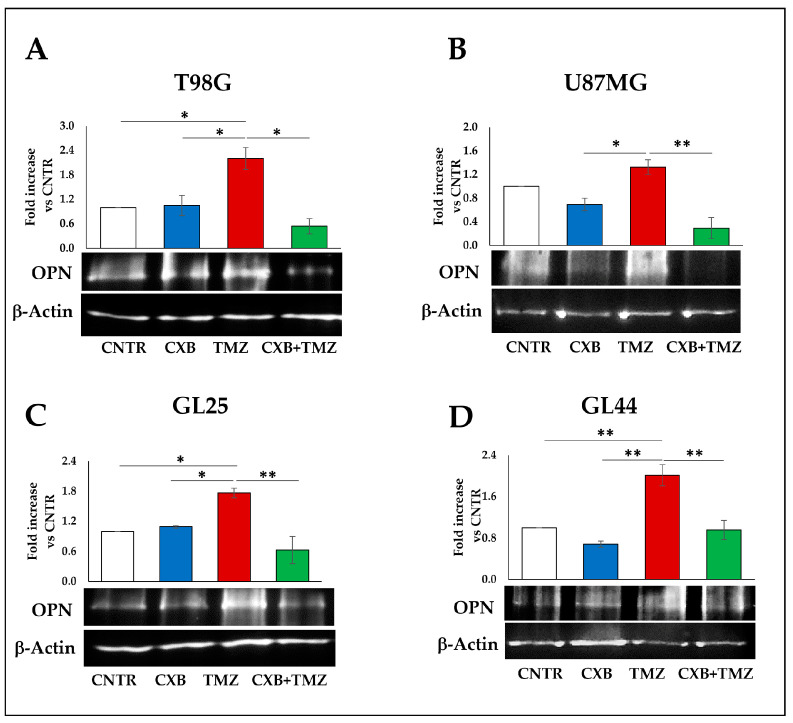
COX-2 inhibition counteracted TMZ-induced OPN overexpression. OPN immunoblotting assays were performed on T98G, U87MG (**A**,**B**), and primary cultures (**C**,**D**) stimulated for 72 h with CXB, TMZ, or their combination and cultured in GSC medium with macrophages. β-Actin was used as a loading control for normalization. Representative images are shown (OPN predicted band size 75 kDa, observed band size ~110 kDa, β-Actin 42 kDa). Values are expressed as the fold increase versus CNTR (mean ± SEM) of three independent experiments. A one-way ANOVA with a post hoc Tukey’s test was used (* *p* < 0.05, ** *p* < 0.01).

Representative Western blot images and the results from the densitometric analysis of OPN levels in GBM spheres from CXB-, TMZ-, and (CXB+TMZ)-treated cells are shown in Figure 6. In GBM cell lines, the OPN expression levels of the CNTR were not significantly affected by CXB, while the TMZ upregulated the OPN expression both in TMZ-partially resistant (U87MG) and TMZ-resistant (T98G) cell lines, and this increase was significant for T98G versus CNTR (Figure 6A,B). The combination CXB+TMZ significantly reduced the OPN expression with respect to TMZ alone, thus counteracting the TMZ effect in both cell lines (Figure 6A,B). In GBM primary cultures, GL25 and GL44, a similar trend was observed; in fact, the effect of TMZ, associated with an increase in OPN expression versus CNTR when used alone, was significantly counteracted by the concomitant exposure to CXB (Figure 6C,D).

### 3.4. COX-2 Inhibition Counteracted the TMZ-Induced CD44 Upregulation

**Figure 7:**
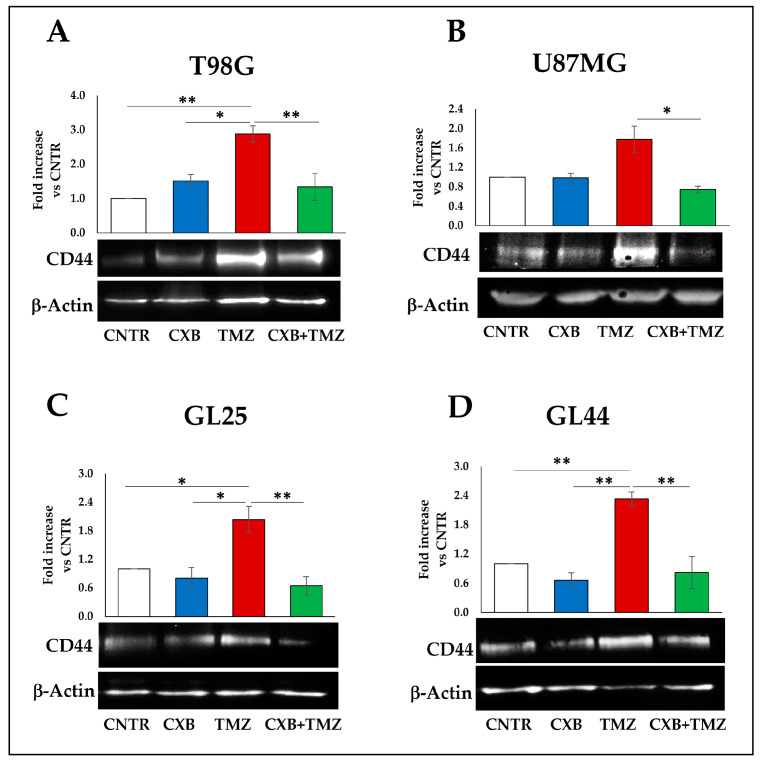
COX-2 inhibition counteracted the TMZ-induced CD44 upregulation. Immunoblotting assays for CD44 were performed on T98G, U87MG (**A**,**B**), and primary cultures (**C**,**D**) previously treated for 72 h with CXB, TMZ, or their drug combination and cultured with macrophages in GSC medium to generate tumorspheres. β-Actin was used as a loading control for normalization. Representative images are shown (CD44 80 kDa, β-Actin 42 kDa). Values are expressed as the fold increase versus CNTR (mean ± SEM) of three independent experiments. A one-way ANOVA with a post hoc Tukey’s test was used (* *p* < 0.05, ** *p* < 0.01).

Through binding with CD44, OPN promotes the stemness phenotype and chemoresistance in glioma [41]. Therefore, we evaluated the effect of the TMZ alone or combined with COX-2 inhibitor on the CD44 expression in GBM spheres derived from cells previously treated with CXB, TMZ, or their combination for 72h, and then cultured with macrophages. As shown in Figure 7A,B, the Western blot analysis of T98G and U87MG revealed a CD44 upregulation after TMZ exposure, even if it was significant versus CNTR only in the T98G cell line. Remarkably, the drug combination was able to drastically reduce the CD44 expression in both cell lines with respect to TMZ (Figure 7A,B). As observed for OPN expression (Figure 6), also for CD44 levels, the primary cultures showed a trend similar to cell lines since TMZ induced an increase in CD44 levels, and the drug combination lowered the CD44 expression to the CNTR levels in both primary cultures (Figure 7C,D).

### 3.5. Effect of Exogenous PGE2 on TMZ-Induced OPN in GBM Cells

**Figure 8:**
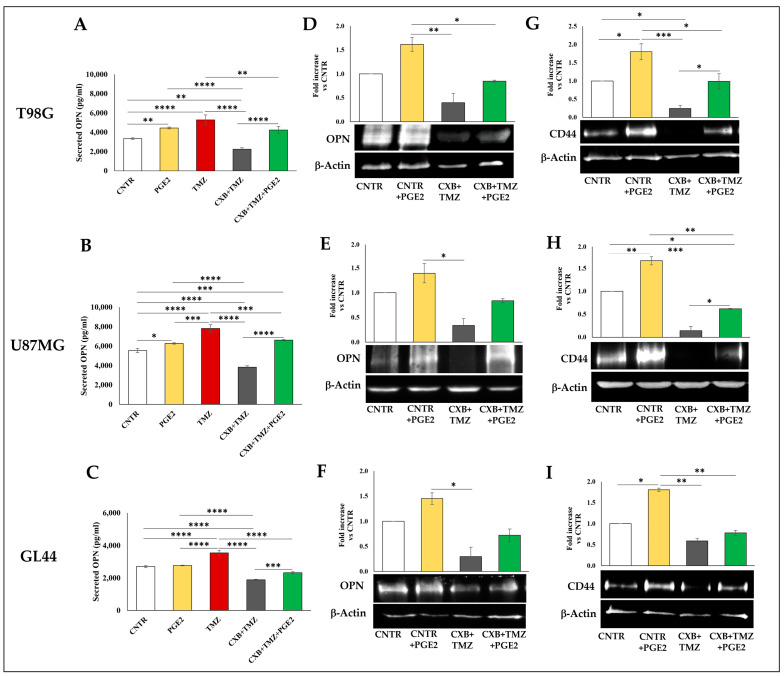
PGE2 contributed to GBM chemoresistance. OPN levels secreted in (**A**) T98G-, (**B**) U87MG-, and (**C**) GL44-sphere supernatants were assessed by ELISA upon stimulation with exogenous PGE2 and the drug combination CXB+TMZ for 72 h in the presence of macrophages. OPN immunoblotting assays of (**D**) T98G, (**E**) U87MG, and (**F**) GL44 treated as described above were normalized vs. β-Actin. CD44 immunoblotting assays of (**G**) T98G, (**H**) U87MG, and (**I**) GL44 treated as described above were normalized vs. β-Actin. Representative images are shown. Values are expressed as the fold increase versus CNTR (mean ± SEM) of two independent experiments. A one-way ANOVA with a post hoc Tukey’s test was used (* *p* < 0.05, ** *p* < 0.01, *** *p* < 0.001, **** *p* < 0.0001).

Aiming to better define the COX-2 role in TMZ resistance, the effect of exposure to exogenous PGE2 was evaluated on OPN and CD44 levels of T98G and U87MG, and GL44 in the presence of macrophages. Since primary cultures showed a similar trend, we selected GL44 to evaluate the effect of exogenous PGE2. The PGE2 addition significantly enhanced the secreted OPN in T98G and U87MG but not in GL44 compared to CNTR (Figure 8A–C). Interestingly, in T98G, U87MG, and GL44, the exogenous PGE2 significantly enhanced the OPN levels in CXB+TMZ-treated cells (green bar) with respect to the drug combination treatment (grey bar) (Figure 8A–C). A similar trend was observed in Western blotting: PGE2 alone strongly enhanced the OPN protein levels, and when added to CXB+TMZ-treated cells, a re-established expression of OPN was detected in GBM cell lines as primary culture even if it was not significant (Figure 8D–F). Additionally, exogenous PGE2 upregulated the CD44 expression compared to CNTR and significantly counteracted the effect of the drug combination in T98G and U87MG spheres (Figure 8G,H). A lower cellular response to the PGE2 addition in CD44 expression was observed in GL44 cells (Figure 8I).

## 4. Discussion

TMZ resistance is an important limitation for treating GBM, one of the most aggressive cancers. Generally, chemoresistance is strongly influenced by the complex interactions of cancer cells and highly tumorigenic GSCs with TME cell components, particularly macrophages. Increasing evidence has demonstrated the role of GAMs in GBM resistance. In the TME, a pro-tumor M2 macrophage polarization is promoted and sustained by a marked increase in immunosuppressive cytokine IL-10, which induces cell growth by activating JAK/STAT3, a COX-2 inductor signaling pathway [42].

COX-2, the enzyme responsible for PGE2 production, is highly upregulated in GBM, and is associated with tumor growth, poor prognosis, and the ability to mediate pleiotropic effects that support proliferation, angiogenesis, and immunosuppression [43]. COX-2 and PGE2 are produced by microglia and macrophages, and PGE2 in the TME is linked to an increased expression of glioma-derived monocyte chemoattractant CCL2/MCP-1, leading to the active recruitment of TAMs [44]. Previous studies have pointed out that the COX-2/PGE2 signaling pathway significantly contributes to the M2 macrophage polarization [45]. In particular, the M2 phenotype is promoted on macrophages by PGE2 after activating the E-series of prostaglandin receptors (EP). About the mechanism involved, the activation of PI3K/Akt signaling by EP receptors is considered a central node for inducing M2 macrophage polarization after COX-2 activation [46,47]. If the COX-2/PGE2 axis is responsible for M2 shifting, its inhibition can effectively counteract the TMZ effects associated with its ability to upregulate COX-2, including the phenotypic shift of macrophages towards M2. The PI3K/Akt signaling block by the COX-2 inhibitor or COX-2 gene silencing by siRNA could be an interesting aspect to study also in our models.

The current study aimed to investigate mechanisms underlying TMZ resistance by evaluating the TMZ-induced COX-2 ability to affect the stemness potential and modulate the TME of GBM. To this purpose, CXB, a COX-2 inhibitor, combined with TMZ, was used on adherent cells subsequently cultured in GSC medium in the presence of macrophages. It is known that chemotherapy, increasing the chemoattractant factor production, strongly induces monocyte recruitment into the tumor which differentiate into M2 and suppress anti-tumor immunity [48]. Here, further deepening our previous data, we evaluated the CXB and TMZ combination on the stemness potential of GBM cells and also on the functional heterotypic interaction of human macrophage cell line U937 with human GBM spheres. We show evidence that TMZ improved the adherent cell’s ability to generate tumorspheres as well increase macrophage infiltration. Surprisingly, tumorspheres derived from TMZ-treated GBM cells released higher amounts of TGF-β1 and IL-10 and were featured by a higher percentage of CD206-positive cells, supporting a macrophage polarization towards the M2 phenotype and, thus, an immunosuppressive and pro-tumor microenvironment. Of note, the COX-2 inhibition, combined with the chemotherapy drug, effectively counteracted the effect of TMZ both at the level of tumorsphere growth and macrophage infiltration. Also, the increase in IL-1β secretion along with the simultaneous decrease in TGF-β1, IL-10, and CD206 levels suggest that the COX-2 inhibition could redirect macrophages towards a pro-inflammatory, anti-tumor M1 phenotype, opposing the effects of TMZ.

To the best of our knowledge, this is the first study showing the ability of TMZ to induce the COX-2 level increase in the tumorsphere model and the promotion of the immunosuppressive microenvironment in the context of resistant GBM cells. These effects seem to be mediated by the TMZ ability to upregulate COX-2; in addition, the inhibition of this enzyme counteracted the TMZ-induced effects.

New strategies to counteract the establishment of a GBM immunosuppressive TME are aimed at repolarizing M2 to the M1 phenotype and reducing the recruitment of tumor-promoting macrophages by targeting chemoattractant molecules such as OPN. Moreover, OPN silencing in human GBM primary cultures significantly reduced macrophage recruitment, sensitizing them to CD8+T cell killing and improving the survival of glioma-bearing mice [11]. Also, OPN has been associated with drug resistance in several cancer types since it is overexpressed in tumor stem cells, crucial players in resistance [49,50]. OPN, via the activation of the CD44 receptor, supports the GBM spheres’ growth and tumorigenicity by the involvement of the PI3K/Akt/mTOR pathway [20,21]. OPN/CD44 crosstalk activation has been reported to promote the stemness phenotype and radioresistance [41]. Moreover, OPN silencing by siRNA enhanced the TMZ-induced apoptosis in U251MG cells and repressed the TMZ-induced NF-κB activation [25].

About the link COX-2/OPN, OPN via α9β1integrin receptor activates the p38 and ERK signaling pathways which upregulate COX-2 expression and activity in tumor-associated macrophages, leading to enhanced angiogenesis and tumor growth [51]. It has been demonstrated that CXB significantly suppressed the ability of OPN to affect human prostatic carcinoma cell line (PC-3) migration [50]. Also, in the xenograft model, mice fed with CXB showed an evident reduction in OPN-induced tumor growth [52]. On the other hand, the COX-2 inhibitor can downregulate OPN levels; the mechanism underlying this effect could be the blockade of NR4A2 (nuclear receptor subfamily 4, group A, member 2) and Wnt/β-catenin signaling, important components involved in OPN regulation [53,54].

In the present work, the TMZ treatment of adherent cells positively affected the OPN and CD44 release in macrophage-infiltrated tumorspheres, sustaining the pro-tumorigenic status. COX-2 inhibition once again significantly reverted the TMZ effect. In particular, the COX-2 inhibition, in a drug combination approach with TMZ, reduced the stemness potential and hindered tumorspheres’ macrophage recruitment, affecting the GBM microenvironment. Interestingly, when COX-2 was inhibited, the exogenous PGE2 addition to adherent cells weakened the drug combination effect, being able to increase the expression of OPN and CD44 in macrophage-infiltrated tumorspheres. This trend has been observed in all cellular models, even if in the GL44 primary culture it was not statistically significant. Overall, despite the higher heterogeneity of primary cultures compared to cell lines, the trend of the various parameters observed in our experimental conditions is similar between cellular models, even if not always accurately specular.

Emerging research has demonstrated that, paradoxically, chemotherapy can actively induce changes supporting tumor progression and resistance. In GBM, TMZ, despite being a cornerstone treatment with a hopeful initial response, is a critical factor that causes resistance in most patients, which quickly relapses. Even if further and more wide-ranging studies are needed to deepen the effects of the COX-2 inhibitor on the TMZ ability to affect the TME in the context of GBM, our data emphasize the paradoxical and alarming pro-tumor effect of TMZ, a treatment that while inducing the recruitment of macrophages, promoted their M2-phenotypic shift, counteracting its efficacy and enriched tumor population with GSCs, resulting in a drug resistance increase. The most important contributor to TMZ resistance is MGMT, which can counteract DNA alkylation damage induced by TMZ. The high methylation status of the MGMT gene promoter region, which may change throughout treatment, results in a decreased expression of MGMT protein correlating with a prolonged survival in GBM patients [55,56,57]. However, low MGMT levels (deficiency and low expression) are still sufficient to confer resistance to TMZ [58], suggesting the existence of MGMT-independent mechanisms, such as enhanced antioxidant systems that contribute to the acquired TMZ resistance [59].

## 5. Conclusions

The published data support the notion that TAM could be a target whose function can be pharmacologically influenced to prevent its recruitment and/or pathological activation in the TME. In particular, a deeper understanding of the COX-2 functional role in an immunosuppressive TME could open up new, targeted, and more effective therapeutic approaches beyond those based on TMZ. Here, the collected results emphasize the paradoxical role of TMZ that counteracts itself efficacy by increasing COX-2 levels and highlight the crucial role of the COX-2/PGE2/OPN axis as an attractive and potent therapeutic target for GBM treatment. Experiments of COX-2 gene silencing, aimed at understanding the contribution of TMZ-induced COX-2 in resistance mechanisms, are currently ongoing. Moreover, our next goal will be evaluating the drug combination effect on macrophage infiltration on organoids, complex 3D cell structures that better mimic the TME, and macrophage-infiltrated organoids, to better define the COX-2 impact in the TMZ-resistance mechanism.

## Figures and Tables

**Table 1:** List of primary antibodies used in the present study.

Primary Antibody	Dilution	Company
rabbit monoclonal anti-COX-2	1:1000	Cell Signaling Technology, Danvers, MA, USA
rabbit monoclonal anti-osteopontin	1:1000	Boster Biological Technology, Pleasanton, CA, USA
mouse monoclonal anti-CD44	1:1000	Cell Signaling Technology, Danvers, MA, USA
mouse monoclonal anti-β-actin	1:1000	Bio-Rad, Hercules, CA, USA

## Data Availability

The data that support the findings of this study are available from the corresponding author upon reasonable request.

## Supplementary Materials

The following supporting information can be downloaded at https://www.mdpi.com/article/10.3390/cells13030258/s1, Figure S1: Stemness potential of GBM primary cultures; Figure S2: COX-2 basal expression of GBM primary cultures; Figure S3: Tumorsphere formation ability of GBM cells following exposure to CXB, TMZ, and their combination; Figure S4: Effect of TMZ on GBM primary culture cell viability.
