# Supplementary material for: Involvement of Cyclooxygenase-2 in Establishing an Immunosuppressive Microenvironment in Tumorspheres Derived from TMZ-Resistant Glioblastoma Cell Lines and Primary Cultures
Source: Cells. 2024 Jan 30;13(3):258. doi: 10.3390/cells13030258 (PMC10854914; doi:10.3390/cells13030258)
Supplement: Supplementary file 1 [file cells-13-00258-s001.zip › cells-2727537-supplementary.pdf]

Supplementary material:

Involvement of cyclooxygenase-2 in establishing an immunosuppressive microenvironment in tumorspheres derived from TMZ-resistant glioblastoma cell lines and primary cultures

Francesca Lombardi, Francesca Rosaria Augello, Serena Artone, Alessia Ciafarone, Skender Topi, Maria Grazia Cifone, Benedetta Cinque, Paola Palumbo.

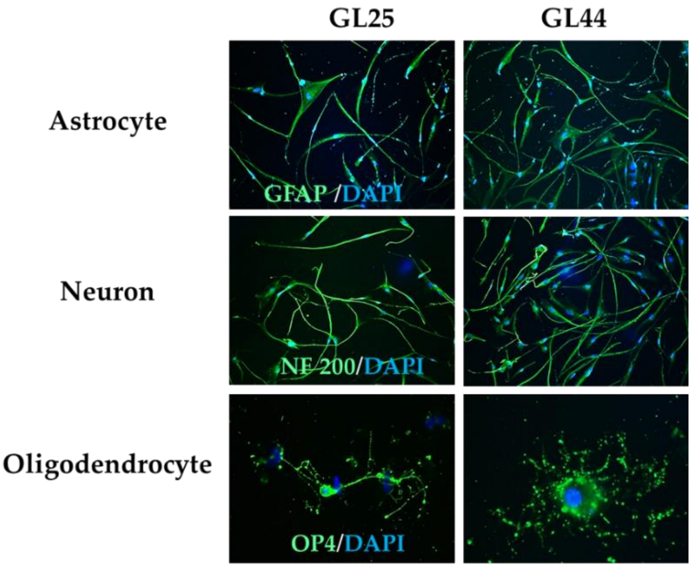

Figure S1: Stemness potential of GBM primary cultures. GL25 and GL44 primary cultures were grown in differentiation NeuroCult™ Neural Cell culture medium for 25 days. Immunofluorescence staining of GFAP (Glial fibrillary acidic protein, astrocyte's marker), NF200 (Neurofilament proteins) and O4 (Oligodendrocyte marker) expression was performed. Nuclei were counterstained with DAPI (blue). Images were acquired at 10× magnification.

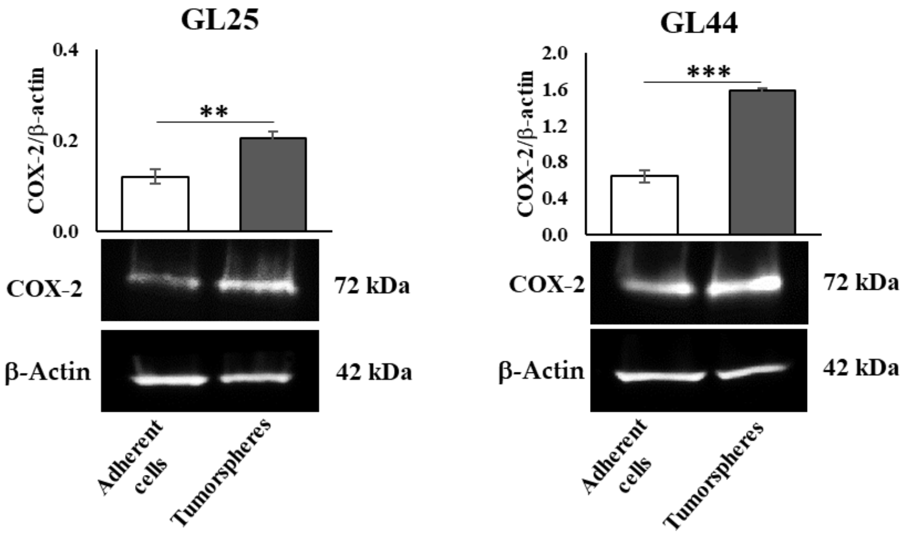

Figure S2: COX-2 basal expression of GBM primary cultures. Basal levels of COX-2 protein measured by western blotting in two GBM primary cultures, GL25 and GL44 respectively, cultured as adherent cells and in tumorspheres' complete medium. The results of the densitometric analysis of bands expressed as ratio vs β-Actin (loading control) band intensity are shown. Data are representative of three independent experiments (mean values ± SEM). One-way ANOVA followed by Dunnett's post-hoc test was used (**P<0.01, ***P<0.001).

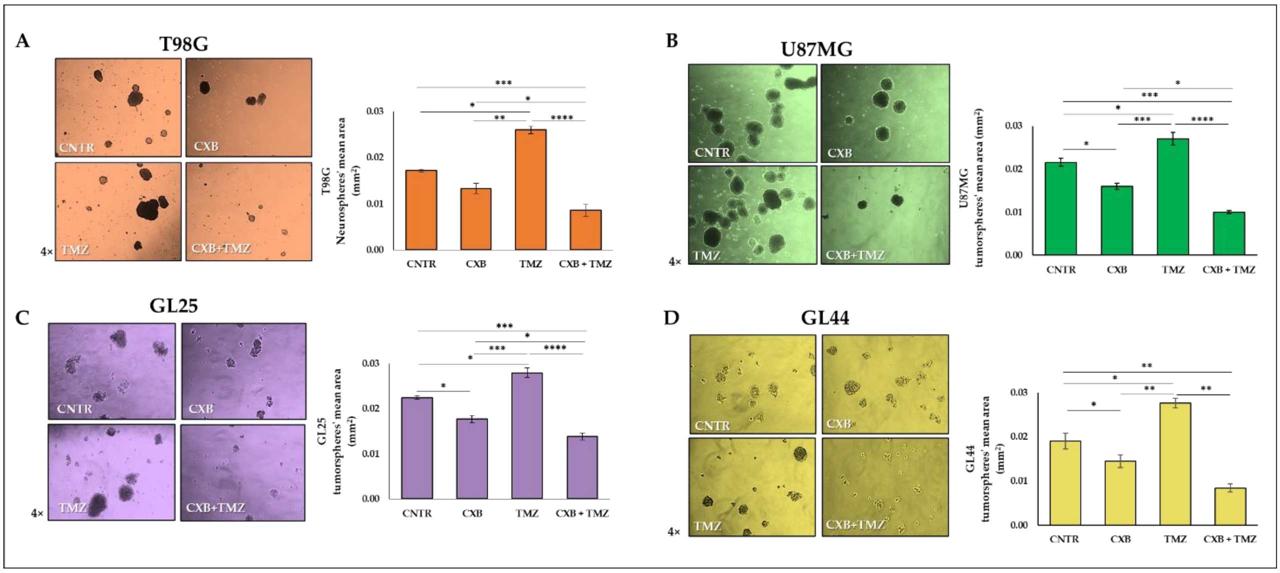

Figure S3: Tumorspheres formation ability of GBM cells following exposure to CXB, TMZ and their combination. Adherent T98G, U87MG, and primary cultures (GL25, GL44), were previously exposed for 72 h to CXB (50 μM), TMZ (200 μM) or combination until spheres' formation and mean area was measured. Representative phase-contrast images and analysis of tumorspheres' mean area were showed. The results are representative of three independent experiments (mean ± SEM). One-way ANOVA followed by Dunnett's post-hoc test was used (* P<0.05, **P<0.01, ***P<0.001, ****P<0.0001).

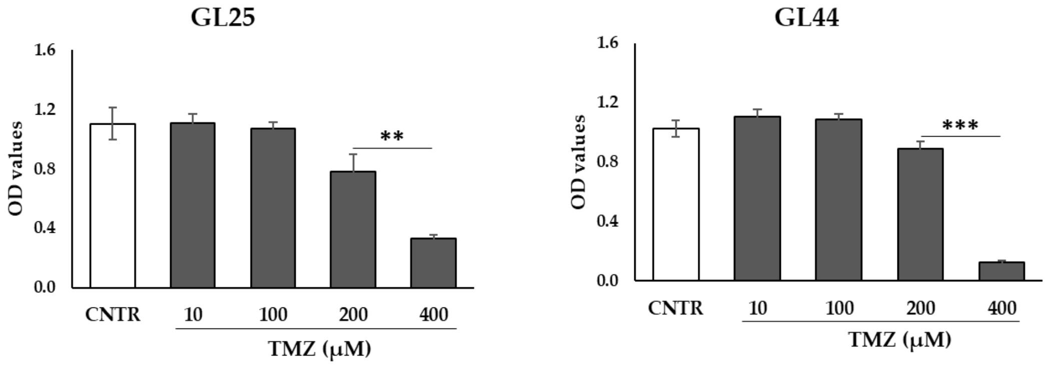

Figure S4: Effect of TMZ on GBM primary cultures cell viability. Cell viability of GL25 and GL44 after 72 h exposure to increasing concentrations (10–400 μM) of TMZ was assessed by CCK8 assay. Data from two independent experiments in triplicate are expressed as mean ± SEM. One-way ANOVA followed by Tukey post-hoc test was used (**P<0.01, ***P<0.001 vs. CNTR).
